# Silicon-on-Insulator (SOI) Lateral Power-Reduced Surface Field FinFET with High-Power Figure of Merit of 239.3 MW/cm^2^

**DOI:** 10.3390/mi16101080

**Published:** 2025-09-24

**Authors:** Chang Woo Song, Taeeun Lee, Dongyeon Kim, Sinsu Kyoung, Sola Woo

**Affiliations:** 1 Department of Electronics and Communications Engineering, Pukyong National University, 45 Yongso-ro, Nam-gu, Busan 48513, Republic of Korea; ckddn4240@pukyong.ac.kr (C.W.S.);; 2 Department of Intelligent Robot Engineering, Pukyong National University, 45 Yongso-ro, Nam-gu, Busan 48513, Republic of Korea; prayer1@pukyong.ac.kr; 3 PowerCubeSemi, Inc., 686, Cheonggyesan-ro, Sujeong-gu, Seongnam-si 13105, Republic of Korea

**Keywords:** reduced surface field (RESURF), silicon on insulator (SOI), lateral power MOSFET, breakdown voltage (BV), specific on-resistance (R_on,sp_)

## Abstract

In this study, we propose a lateral power-reduced surface field FinFET (LPR-FinFET) to achieve high breakdown voltage and low on-resistance. We investigate the electric field distribution within the reduced surface field (RESURF) structure under reverse-biased conditions, as well as forward transfer and output characteristics using TCAD simulation. The proposed LPR-FinFET demonstrates a high breakdown voltage of 247 V and a low specific on-resistance of 0.255 mΩ·cm^2^ with a high-power figure of merit of 239.3 MW/cm^2^. The superior characteristics of our proposed LPR-FinFET show the potential for applications as a lateral power semiconductor using silicon-on-insulator (SOI) technology.

## 1. Introduction

In recent years, the demand for efficient and reliable power semiconductor devices has significantly increased due to the rapid growth of various high-power applications, such as electric vehicles, renewable energy systems, and advanced communication technologies [1,2,3,4]. While emerging technologies focusing on wide-bandgap materials such as silicon carbide (SiC) and gallium nitride (GaN) have gained significant attention in recent years, silicon-based power devices continue to dominate the market due to their maturity, cost-effectiveness, and well-established manufacturing processes [5,6,7,8,9,10]. Among them, silicon-on-insulator (SOI) lateral power devices have been widely utilized in power-integrated circuits as a promising solution to address the limitations of traditional bulk silicon devices due to their high breakdown voltage, outstanding insulation properties, and low power consumption [11,12,13,14]. Although extensive studies have been conducted to improve the breakdown voltage (BV) of SOI lateral power devices (including techniques like junction termination extension (JTE) and reduced surface field (RESURF)) [15,16,17], there remains an issue where the on-state performance degrades as BV increases due to the trade-off between BV and on-resistance.

In addition, most prior research on power FinFETs has focused on vertical architectures for high-voltage applications. In contrast, studies on lateral power FinFETs are limited and primarily include structures such as superjunction (SJ) FinFETs and high-k passivation FinFETs. For example, Yoo et al. [17,18] and Onishi et al. [19] proposed lateral SJ-FinFETs using deep trench or P/N pillar configurations to enhance BV. However, these approaches introduce complex fabrication steps and tight doping control requirements, which hinder practical scalability. In particular, their breakdown voltage (BV) remained limited to around 100 V and the specific on-resistance (R_on,sp_) was as high as 1 mΩ·cm^2^ [17,18,19]. Cheng et al. [11], on the other hand, demonstrated a lateral Fin MOSFET with high-k dielectric passivation between the gate and drain to enhance BV. However, in practice, the high-k/Si interface often leads to trap formation and electric field crowding, resulting in increased R_on,sp_ and reduced BV.

To address these limitations, we propose a novel lateral power FinFET structure utilizing a multi-RESURF technique that introduces an additional *p*-type top layer (*p*-top) in the drift region. To the best of our knowledge, such a multi-RESURF FinFET using a top layer has not been previously reported for lateral power devices. This structure effectively redistributes the electric field near the gate edge, allowing for high BV while maintaining a low R_on,sp_—without relying on high-k materials or complex SJ architectures.

In this paper, we demonstrate the design process of lateral power-reduced surface field FinFET (LPR-FinFET) devices and present their electrical characteristics using Sentaurus TCAD simulations. Additionally, we calculate BV and R_on,sp_ under various conditions to optimize the trade-off between them.

## 2. Materials and Methods

**Figure 1:**
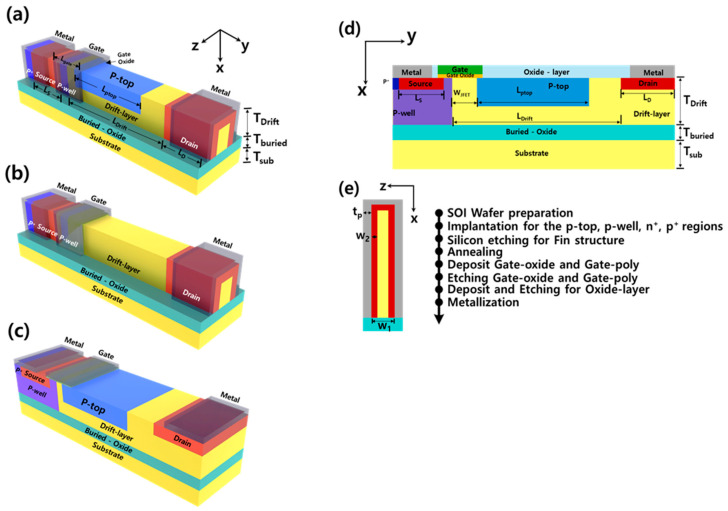
Three-dimensional structure of (**a**) LPR-FinFET, (**b**) LP-FinFET, and (**c**) conventional planar MOSFET. (**d**) Two-dimensional structure of LPR-FinFET (XZ plane). (**e**) Brief process flow chart of LPR-FinFET.

Figure 1a–c present a 3D schematic view of the proposed LPR-FinFET, LP-FinFET, and conventional planar MOSFET structures, and Figure 1d,e illustrate cross-sectional views from the XY plane and XZ plane, respectively. The proposed device is designed on an SOI substrate, which consists of a buried oxide layer between the bottom substrate and the top drift layer. The top drift layer is doped with a *p*-well and a *p*-top. The top drift layer has a doping concentration of 2.5 × 10^16^ cm^−3^. The *p*-well and *p*-top regions have a doping concentration of 1 × 10^17^ cm^−3^ and 8 × 10^15^ cm^−3^, respectively. Additionally, the *p*^+^, *n*^+^ source (1 × 10^19^ cm^−3^), and *n*^+^ drain (1 × 10^19^ cm^−3^) regions are doped with boron and arsenic. In this study, the JFET region width (W_JFET_) was fixed at 2.0 μm to focus on the effects of the *p*-top parameters. The top drift layer is covered by a fin-shaped structure consisting of a gate, oxide layer, and metal. The application of RESURF techniques with a *p*-top layer can adjust the surface electric field, effectively reducing the rate of impact ionization. In the proposed device, the *p*-top region with a RESURF structure is expected to provide field spreading effects and enhance BV of the device. Therefore, in terms of device characteristics such as BV and R_on,sp_, controlling the dose and length of the *p*-top region and the drift-layer is critical. Accordingly, this work focuses on investigating the impact of varying the *p*-top dose and length on the device characteristics. Other structural parameters, such as the drift layer thickness (T_drift_) and the buried oxide thickness (T_buried_), were verified to have negligible impact on BV and R_on,sp_ within the considered design space. Therefore, their values are fixed, as shown in Table 1, to reduce the complexity of the design space and focus on the dominant factors affecting performance. Figure 1d illustrates a brief process flow chart for the LPR-FinFET using the conventional CMOS process.

The proposed device was designed using Sentaurus TCAD (version V-2023.12). The detailed parameter values of the device were taken from previous research [10,11] and are presented in Table 1. To evaluate the electrical characteristics of the device, device simulations were carried out with the high-field-saturation model, Schottky-Read-Hall recombination model, Auger recombination model, and Avalanche model (vanOverstraeten). All TCAD simulations were performed at a fixed temperature of 300 K under DC operating conditions to provide a clear baseline comparison of device structures. For simplicity, the *p*-top region was modeled with abrupt junctions without including diffusion effects.

## 3. Results and Discussion

The RESURF effect in LPR-FinFET introduced by the *p*-top region enables better electric field distribution and charge depletion, allowing for a higher drift doping concentration and lower R_on,sp_ without sacrificing BV. This improves the BV-R_on,sp_ trade-off beyond the conventional silicon limit [15]. 

### 3.1. Forward Characteristics

**Figure 2:**
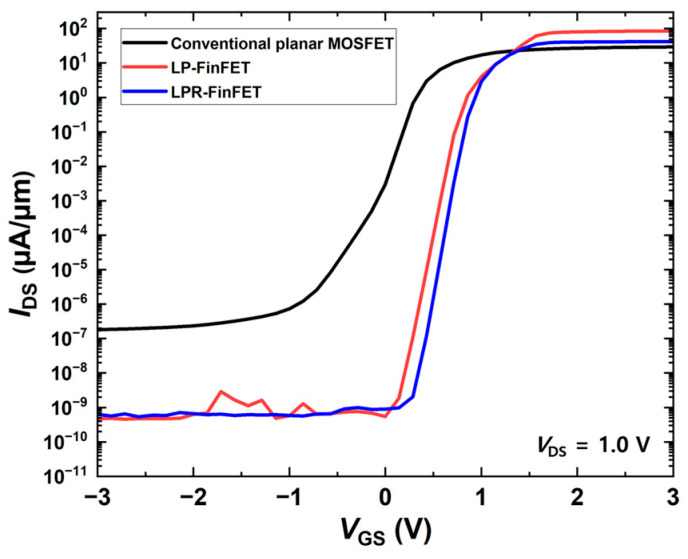
Comparison of the forward transfer characteristics of conventional planar MOSFET, LP-FinFET, and LPR-FinFET at *V*_DS_ = 1.0 V.

Figure 2 shows the forward transfer characteristics (*I*_DS_-*V*_GS_) of the conventional planar MOSFET, conventional lateral power FinFET (LP-FinFET), and LPR-FinFET at *V*_DS_ = 1.0 V. The transfer characteristics indicate that the 3-D structures, which are the FinFET structures, exhibit a higher drain current (*I*_DS_) than the conventional planar structure. To extract the threshold voltage, we used the constant current method with a drain current of 1 μA/μm as a reference. The threshold voltages for the conventional planar MOSFET, LP-FinFET, and LPR-FinFET are 0.323 V, 0.847 V, and 0.922 V, respectively, while the leakage currents are ~10^−13^ A, ~10^−16^ A, and ~10^−16^ A, respectively. Both the LP-FinFET and LPR-FinFET exhibit higher threshold voltages and lower leakage currents than the conventional planar MOSFET, indicating reduced power consumption in the off-state. Although a lower threshold voltage can improve switching speed, the conventional planar MOSFET shows a low *V*th (0.323 V) together with high leakage, a small on/off current ratio, and a poor subthreshold slope. By contrast, the LP-FinFET and LPR-FinFET exhibit moderately higher *V*th (0.847 V and 0.922 V), which effectively suppresses leakage and ensures stable off-state performance.

**Figure 3:**
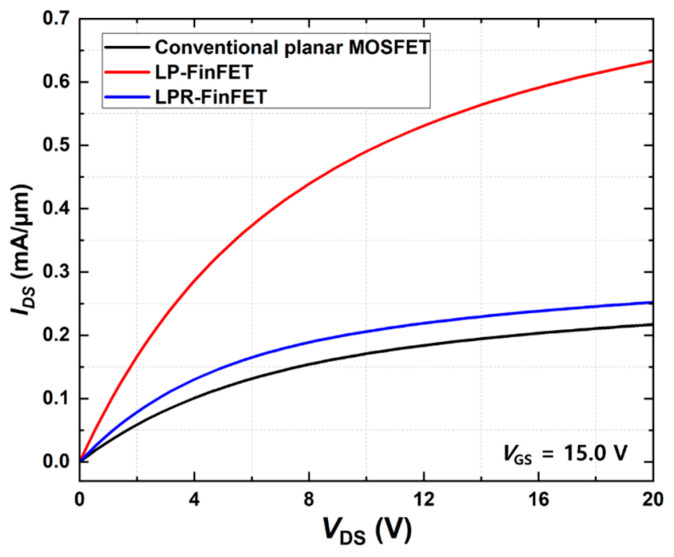
Comparison of the forward output characteristics of conventional planar MOSFET, LP-FinFET, and LPR-FinFET at *V*_GS_ = 15.0 V.

Figure 3 displays the forward output characteristics (*I*_DS_-*V*_DS_) of the conventional planar MOSFET, LP-FinFET, and LPR-FinFET with *V*_GS_ = 15 V. The 3D FinFET structures show higher drain current (*I*_DS_) compared to the conventional planar design. To calculate the on-state resistance (R_on,sp_) of each device, the slope in the linear region (*V*_DS_ = 0.0 V to 2.0 V) was extracted. The R_on,sp_ values are 0.340 mΩ·cm^2^ for the conventional planar MOSFET, 0.120 mΩ·cm^2^ for the LP-FinFET, and 0.255 mΩ·cm^2^ for the LPR-FinFET.

Both LP-FinFET and LPR-FinFET exhibit reduced R_on,sp_ due to the increased effective channel area of the fin structure, which improves current flow in the on-state. Although LP-FinFET, in comparison to other devices, demonstrates the highest drain current and the lowest R_on,sp_, it is expected to have lower BV characteristics than LPR FinFET due to the electric field concentration phenomenon caused by the absence of RESURF techniques. Therefore, an optimal design for LPR-FinFET with the RESURF structure is essential to minimize R_on,sp_ while maximizing the BV.

### 3.2. Electric Field Distributions

**Figure 4:**
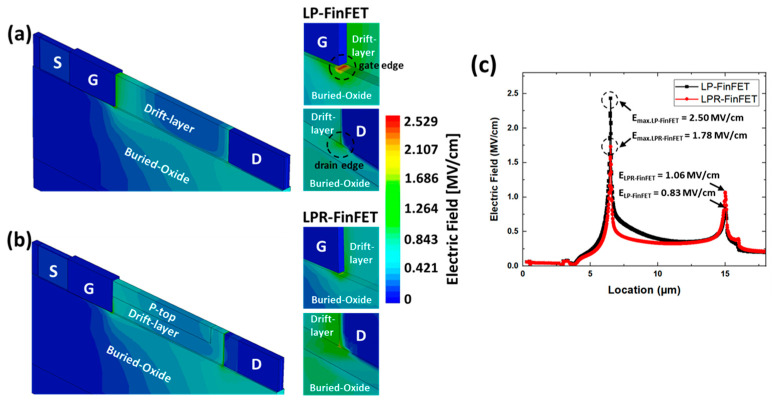
Three-dimensional view of electric field distribution of (**a**) LP-FinFET and (**b**) LPR-FinFET (L_ptop_ = 8 μm, N_ptop_ = 8 × 10^15^ cm^−3^) at *V*_DS_ = 300 V and *V*_GS_ = 0 V. (**c**) Electric field distribution for (**a**,**b**).

Figure 4a,b show the electric field distribution in the conventional LP-FinFET and the proposed LPR-FinFET at *V*_DS_ = 300 V, respectively. In LPR-FinFET, the *p*-top length (L_ptop_) was set to 8 μm and the doping concentration (N_ptop_) was set to 8 × 10^15^ cm^−3^. As shown in Figure 4a, the LP-FinFET exhibits an electric field concentration at the gate edge, which can lead to degradation of device performance such as the breakdown voltage. To reduce the concentrated electric field at the gate edge, the *p*-top was applied in LPR-FinFET to distribute the concentrated electric field. Figure 4b shows the reduced electric field at the gate edge in LPR-FinFET at the same voltage, *V*_DS_ = 300 V, indicating improved electric field distribution using the RESURF techniques. For a more detailed analysis, Figure 4c provides a comparison of the electric field distribution from the source to drain for both devices. The electric field profiles in Figure 4 are obtained at *V*_DS_ = 300 V, which exceeds the breakdown voltages of both devices, in order to clearly compare the peak field characteristics under extreme bias. The field in Figure 4c is extracted along a horizontal line just beneath the gate oxide, where lateral field peaks occur. The maximum electric field (E_max_) occurs at the gate edge in both cases, with E_max_ = 2.50 MV/cm for LP-FinFET and E_max_ = 1.78 MV/cm for LPR-FinFET, representing a 28% reduction in LPR-FinFET. Additionally, the higher electric field value observed near the drain edge of the LPR-FinFET structure indicates that the electric field is redistributed from the gate edge towards the drift layer near the drain edge. In LPR-FinFET, the *p*-top region contributes to electric field redistribution by suppressing the field near the gate edge and shifting it toward the drain. This lateral spreading of the field improves breakdown characteristics by avoiding excessive field concentration at a single point. It indicates that the *p*-top effectively redistributes the concentrated electric field at the gate edge. Therefore, the *p*-top region expands the lateral depletion width and smooths the electric field profile across the drift region, which suppresses the peak field near the drain junction. This reduces the likelihood of impact ionization and contributes to an enhanced breakdown voltage.

### 3.3. Breakdown Voltage Characteristics

**Figure 5:**
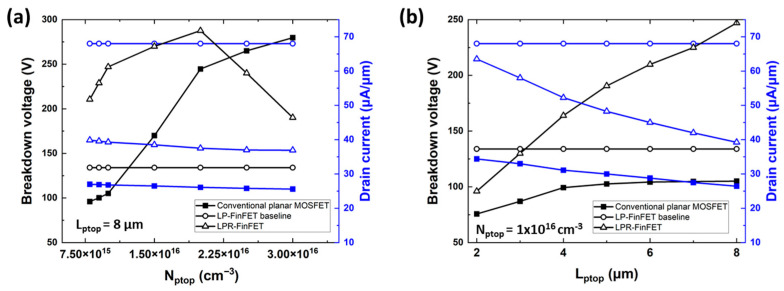
Breakdown voltage (BV) characteristics and drain current of conventional planar MOSFET and LPR-FinFET depending on (**a**) *p*-top doping concentration and (**b**) *p*-top length.

Figure 5a,b depict the breakdown voltage and on-state drain current of the conventional planar MOSFET and LPR-FinFET as a function of *p*-top length (L_ptop_) and doping concentration (N_ptop_), respectively. As N_ptop_ increases from 8 × 10^15^ cm^−3^ to 3 × 10^16^ cm^−3^, the drift layer forms a wider depletion region to achieve charge balance, allowing for more effective electric field distribution with high BV, as shown in Figure 5a. This indicates that a higher voltage is required to reach the critical electric field (E_c_), resulting in an increase in the breakdown voltage. However, the higher N_ptop_ slightly reduces the on-state drain current due to the expansion of the depletion region.

Furthermore, increasing the *p*-top doping concentration beyond an optimal point does not continuously improve BV; rather, it can cause a reduction in BV. Excessive doping concentrations beyond this optimal level disrupt the charge balance, leading to localized high electric fields, which lower the BV in the LPR-FinFET when N_ptop_ exceeds 2.25 × 10^16^ cm^−3^, as shown in Figure 5a.

As shown in Figure 5b, as L_ptop_ increases from 2 μm to 8 μm, the *p*-top region extends, allowing the total voltage to be distributed over a larger area. As a result, the electric field is more uniformly distributed across the depletion region, enabling the longer RESURF structure to achieve a higher BV. However, this extension of L_ptop_ also results in a decrease in the drain current due to the expansion of the depletion region. Consequently, the trade-off between BV (which corresponds to off-state performance) and the on-state drain current (which is related to on-state resistance) becomes increasingly apparent as device parameters such as *p*-top length and doping concentration are optimized. This relationship is crucial for balancing the electric field distribution and depletion region expansion to achieve an optimal design that maximizes breakdown voltage while minimizing on-state resistance and drain current degradation in high-power applications.

It should be noted that in practical fabrication, dopant diffusion during thermal processes can broaden the *p*-top profile and alter the effective junction depth. Such diffusion may shift the optimal values of L_ptop_ and N_ptop_, potentially modifying the balance between breakdown voltage and R_on,sp_. While this effect was not included in the present simulations, it represents an important aspect for future process-aware studies. In addition, while dynamic characteristics such as turn-on and turn-off switching times were not directly simulated in this work, the RESURF-based field redistribution in LPR-FinFET is expected to reduce the effective gate-drain capacitance compared to conventional structures, which may positively influence switching performance.

**Figure 6:**
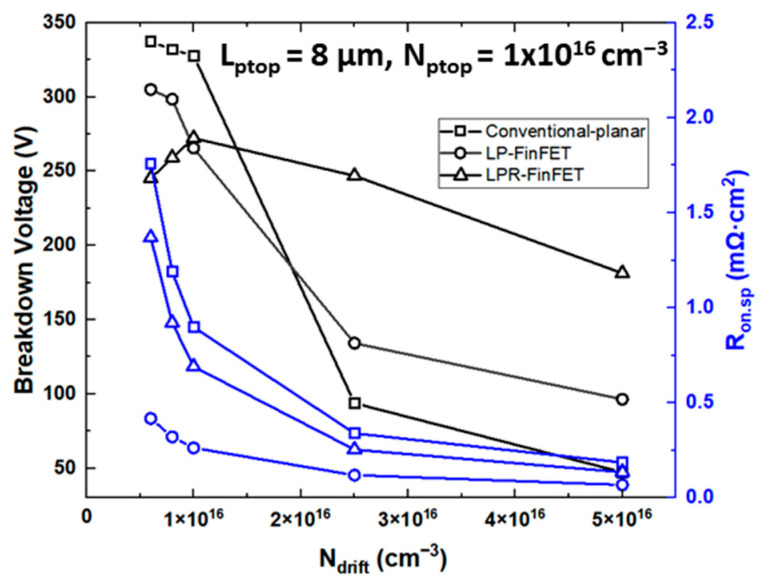
Breakdown voltage (BV) characteristics and R_on,sp_ of conventional planar MOSFET, LP-FinFET, and LPR-FinFET depending on drift doping concentration.

Figure 6 presents the breakdown voltage and specific on-state resistance (R_on,sp_) characteristics of the conventional planar MOSFET, LP-FinFET, and LPR-FinFET structures as a function of drift layer doping concentration (N_drift_). An increase in N_drift_ results in a decrease in BV due to the intensification of the electric field in the drift region, while R_on,sp_ decreases as a result of the higher carrier concentration, as shown in Figure 6. From the analysis of the trade-off between BV and R_on,sp_, it was observed that at N_drift_ = 2.5 × 10^16^ cm^−3^, the devices exhibit minimal variation in R_on,sp_ and relatively stable BV. Consequently, based on both the forward and reverse characteristics, the optimal values of L_ptop_ = 8 μm, N_ptop_ = 1 × 10^16^ cm^−3^, and N_drift_ = 2.5 × 10^16^ cm^−3^ were identified in our simulation results. This configuration achieves sufficient lateral depletion and field spreading to enhance breakdown voltage, while minimizing the degradation in specific on-resistance. Under these conditions, the LPR-FinFET device demonstrated superior BV and R_on,sp_ performance compared to the other devices. This study has been carried out as a simulation-based proof-of-concept, focusing on the electrostatic trade-off between breakdown voltage and on-resistance. The proposed structure can be realized using standard CMOS-compatible processes, which can be utilized in future experimental investigations.

Table 2 presents a summary of the simulated static characteristics for the three devices, including the conventional planar MOSFET, conventional LP-FinFET, and proposed LPR-FinFET. The power figure of merit (P-FOM) is defined as P-FOM = BV^2^/R_on,sp_, where BV is the breakdown voltage and R_on,sp_ is the specific on-resistance [12,13,14,20,21,22,23,24,25]. This metric is commonly used to evaluate the performance trade-off between high breakdown voltage and low on-resistance in unipolar power devices. While this metric is widely used in the literature, it assumes an ideal unipolar behavior where R_on,sp_ ∝ BV^2^. However, in practical RESURF-based structures, this relationship can deviate depending on the design, as noted in [15]. For example, single-RESURF devices may follow R_on,sp_ ∝ BV^2.5^, meaning that PFOM becomes a function of BV. Therefore, P-FOM is used in this work not as an absolute figure of superiority, but as a relative benchmarking metric under consistent silicon-based FinFET conditions. These values are intended for like-to-like comparisons between the proposed and reference structures, rather than across different device technologies or architectures. Using R_on,sp_ and the BV, it is possible to calculate P-FOM [12,13,14], which is as high as 239.3 MW/cm^2^ for the transistor featuring the optimal points.

**Figure 7:**
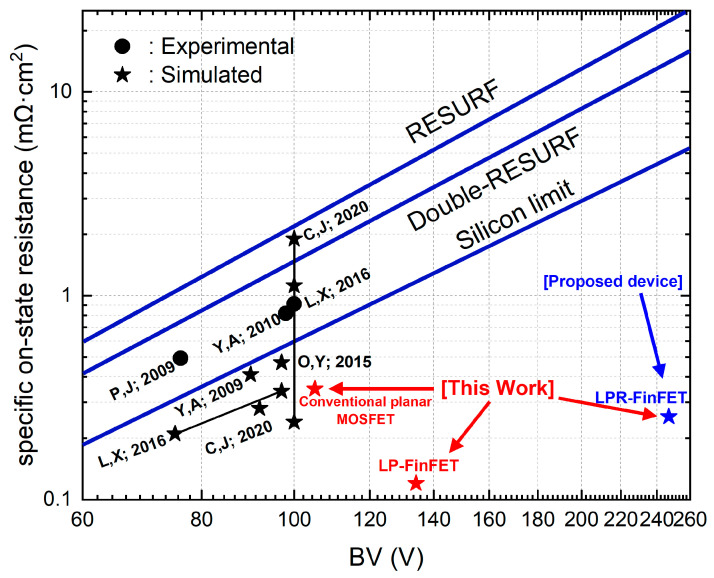
Comparison of the BV-R_on,sp_ relationship between our proposed device and others [7,11,16,17,18,19,26,27].

Figure 7 compares the relationship between R_on,sp_ and BV for our proposed devices and previously reported technologies. For this comparison, we utilized three benchmarks from [15], including RESURF, double-RESURF, and silicon limit. The “silicon limit” describes the empirical relationship between breakdown voltage and specific on-resistance in unipolar silicon devices, typically expressed as R_on,sp_ ∝ BV^2.5^ [15]. This relation sets a material-dependent lower bound that can only be improved by non-uniform doping or electric field shaping, such as in RESURF or superjunction structures. The superior BV-R_on,sp_ performance of the LPR-FinFET, as shown in Figure 7, stems from the inclusion of a *p*-top RESURF region in its drift structure, which fundamentally improves the device’s electric field distribution. The *p*-top layer redistributes the electric field under off-state bias, alleviating the electric field concentration at the gate edge that normally triggers early avalanche breakdown. This field-spreading effect suppresses impact ionization at the gate edge and allows the drift region to support a higher doping concentration without premature breakdown. In other words, the *p*-top layer provides charge balance that widens the depletion region and smooths the field gradient across the drift length, enabling a higher BV even as the drift resistivity is lowered. Consequently, the BV-R_on,sp_ trade-off is shifted in favor of LPR-FinFET: it achieves significantly higher BV with only a slight on-resistance increase compared to the LP-FinFET lacking the *p*-top layer. This design thus represents a much higher P-FOM, even surpassing the traditional “silicon limit” benchmark for lateral power MOSFETs. For instance, the optimized LPR-FinFET reaches a BV of 247 V with R_on,sp_ ≈ 0.255 mΩ·cm^2^ (≈239 MW/cm^2^), versus a BV of 134 V and 0.120 mΩ·cm^2^ (~150 MW/cm^2^) for a comparable LP-FinFET. The inclusion of the *p*-top RESURF structure is therefore key to LPR-FinFET’s enhanced performance, as it modifies the internal electric field landscape to boost BV while minimizing R_on,sp_, yielding a markedly higher P-FOM than previous lateral MOSFET designs [7,11,16,17,18,19,26,27]. Therefore, our proposed LPR-FinFET would be a promising lateral power diffusion device for high-performance power devices.

## 4. Conclusions

In this study, we proposed a novel design methodology for the LPR-FinFET device, utilizing TCAD simulations to optimize the trade-off between BV and R_on,sp_, while mitigating electric field crowding at the gate edge. The proposed LPR-FinFET structure demonstrated superior device performance compared to conventional planar MOSFET and LP-FinFET designs, achieving a P-FOM of 239.3 MW/cm^2^. This high BV and low R_on,sp_ performance represents the potential of the LPR-FinFET as a promising candidate for high-performance power devices, particularly in applications requiring high efficiency and reliability.

## Figures and Tables

**Table 1:** Device parameters for the simulated devices.

Symbol	Meaning	Value
L_drift_	Drift length	12.0 μm
L_D_	Drain length	4.0 μm
L_S_	Source length	3.0 μm
L_ptop_	*p*-top length	2.0~8.0 μm
W_JFET_	JFET region length	2.0 μm
t_p_	Fin Plate thickness	0.1 μm
w_1_	Fin *n*-drift width	0.4 μm
w_2_	N+ width	0.1 μm
T_OX_	Gate oxide thickness	20 nm
T_sub_	Substrate thickness	2.0 μm
T_buried_	Buried oxide thickness	6.0 μm
T_drift_	Drift layer thickness	2.0 μm

**Table 2:** Comparison of optimized device characteristics.

Device	BV (V)	R_on,sp_ (mΩ·cm^2^)	P-FOM (MW/cm^2^)
Conventional-planar	105.1	0.340	32.5
LP-FinFET	134.2	0.120	150.0
LPR-FinFET	247	0.255	239.3

## Data Availability

The original contributions presented in this study are included in the article. Further inquiries can be directed to the corresponding author.
